# Roles of Microbiota in Cancer: From Tumor Development to Treatment

**DOI:** 10.1155/2022/3845104

**Published:** 2022-03-23

**Authors:** Zohreh Bagheri, Leila Moeinzadeh, Mahboobeh Razmkhah

**Affiliations:** ^1^Shiraz Institute for Cancer Research, School of Medicine, Shiraz University of Medical Sciences, Shiraz, Iran; ^2^Department of Tissue Engineering and Applied Cell Sciences, School of Advanced Medical Sciences and Technologies, Shiraz University of Medical Sciences, Shiraz, Iran

## Abstract

Cancer as a second leading cause of death arises from multifactorial pathology. The association of microbiota and their products with various pathologic conditions including cancer is receiving significant attention over the past few years. Mounting evidence showed that human microbiota is an emerging target in tumor onset, progression, prevention, and even diagnosis. Accordingly, modulating this composition might influence the response to tumor therapy and therapeutic resistance as well. Through this review, one could conceive of complex interaction between the microbiome and cancer in either positive or negative manner by which may hold potential for finding novel preventive and therapeutic strategies against cancer.

## 1. Introduction

In patients with local early-stage tumor, surgical resection can be potentially curative; however, it is no longer applicable for patients with metastatic tumor. Even though chemotherapy and immunotherapy, the centerpiece in treatment of late-stage tumors, have proved effective early on, unfortunately all patients will gradually develop resistance to them and suffer from cancer progression. Thus, this would necessitate not only resolving the adaptive resistance of cancerous cells to anticancer components but also finding new alternative treatments to eliminate the disadvantages of conventional treatments. The microbiota recently has received substantial attention given its influence on diverse diseases, in particular cancer. Close to 100 trillion dynamic microorganisms, representing over 5000 various species including bacteria, viruses, fungi, and parasites, inhabit in the human body at numerous sites including the skin and mucosal surfaces of different organs such as the gastrointestinal tract [1, 2]. They have a profound role in different aspects of human physiology such as protecting the body against pathogenic microorganisms, promoting the immune system, and helping in food digestion, absorption, and metabolism [3]. The “microbiota” refers to the collection of settled microbes that are resident on and inside the body, and the “microbiome” is defined as all the genomes of this microbiota [4]. For reasons of interacting with the host all over the life span, it is not unexpected that microorganisms play such a robust role in various functions of host body [1, 5]. In addition to the number, the diversity of these microbes also plays a pivotal role in the formation and maintenance of human health state [4]. In this review, the widespread association between commensal microbiota and cancer is highlighted. In addition, we discuss the role of human microbiota in dysbiosis state on development, screening, and management of tumors.

## 2. Microbiota Diversity

The composition of microbiota is distinctive to each individual and remains relatively unchanged and resilient during the whole adult life span based on genetic features, immune system characteristics, health situation, body mass index, diet pattern, lifestyle, and other environmental factors [5–8]. For instance, administration of fecal microbiota from obese individual to germ-free (GF) mice brings about gaining more weight than similar mice that received fecal microbes from lean persons [9]. Therefore, microbiota equilibrium that is unique for each person appears to be a great importance for a normal physiology in humans [10, 11]. The human microbiota contributes to maintenance of homeostasis in some critical physiological processes including immune system regulation, inflammatory state [12], intestinal permeability [13], energy balance [14], and endocrine hormone secretion [15]. A growing evidence also point to the interactions between the gut microbiota and brain in varieties aspects [16]. For instance, microbiota in the gastrointestinal (GI) tract can modify signaling pathways involved in stress management through reprograming of the hypothalamic–pituitary–adrenal axis (HPA axis) and in turn normalize basal corticosterone levels [17].

## 3. Microbiota Homeostasis and Dysbiosis

**Figure 1:**
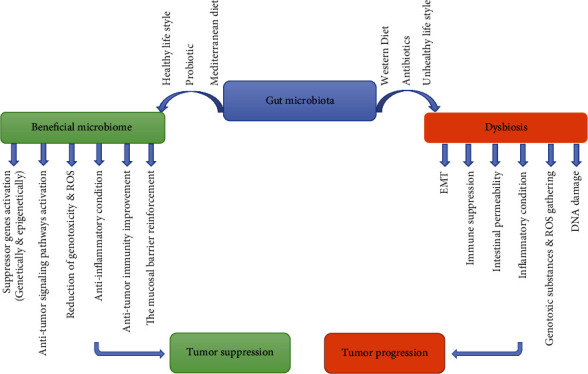
The mechanisms by which carcinogenesis is modulated by microbiota. The most important factors by which microbiota turn to dysbiosis are unhealthy lifestyle, repeated exposure to antibiotics, and Western diet mostly containing animal meat and low fat. Dysbiosis predisposes individuals to certain cancers. Mechanistically, dysbiosis builds protumor environment through EMT induction, antitumor immunity suppression, and intestinal permeability, increases the chance of pathogens entering the bloodstream, inflammation induction, and in turn increase in ROS and genotoxic substances which damage DNA and finally potentiate tumor development. On the other hand, a healthy lifestyle and diet enriched in high fiber and probiotics mediate tumor suppression through raising the level of beneficial microbiome which triggers the reinforcement of mucus barrier, antitumor immunity improvement, inflammation reduction, genotoxic substance clearance, antitumor signaling activation, and genetically and epigenetically tumor suppressor activation.

Both human genetics and environmental factors could influence the homeostasis of microbiota. Plethora data have demonstrated a strong link between homeostasis disturbance of human microbiota, named dysbiosis, and different pathologic conditions ranging from gastrointestinal disturbance, cardiovascular disorders, and neurologic diseases to cancer [18–22]. The comparison between microbiota of patients with colorectal cancer and healthy volunteers demonstrated that there is a statistically substantial difference in bacterial composition between two groups. Among microbiota community, Fusobacteria and Firmicutes showed higher abundance whereas Proteobacteria demonstrated less abundance in these patients. Furthermore, a significant difference in community arrangements of microbiota was obvious in tumor tissues compared to that of adjacent normal tissues. Fusobacterium and Lactococcus were overrepresented while two kinds of bacteria, Pseudomonas and Escherichia-Shigella, were underrepresented in cancerous tissues [23–25]. In a widespread study, plasma-derived microbial nucleic acids of patients with cancer were compared with healthy individuals. The data verified a high discrimination between samples from patients with numerous types of cancer and cancer-free individuals. This disparity has also been observed among various types of cancer, which may endorse the diagnostic value of microbiome profile for cancer in the near future [26]. Results of a preclinical study in which feces of cancer patients were fed to the animals by healthy condition showed that these animals displayed proinflammatory response, local immune alteration, procarcinogenic signal induction, and tumorigenesis which might place great emphasis on the role of microbiota community in cancer development [27–29]. Although underlying mechanisms have not been fully resolved, several studies have been conducted to elucidate mechanisms of dysbiosis-induced cancer and their results pointed to some probably involved mechanisms. It seems that induction of inflammatory microenvironment and epithelial-mesenchymal transition (EMT) are two main molecular mechanisms [30–32]. Increase in reactive oxygen species (ROS) and DNA damage [33], genotoxic substances gathering [34], and suppression of antitumor immune response [35] are considered as other mechanisms in this regard (Figure 1). It is also shown that dysbiosis contributes to cancer development by destruction of the gut mucosal layer and subsequently increased intestinal permeability, raising the translocation of pathogens and its byproducts from the gut and intestine to the other tissues and systemic circulation [36]. The main environmental factors leading to dysbiosis are inappropriate diet pattern [37] and antibiotic consumption (Figure 1) [38].

## 4. The Role of Diet on Microbiota Composition

Diet affects multiple aspects of human health, and inappropriate diet habits undoubtedly contribute to chronic metabolic conditions and development of certain diseases. As mentioned before, one of the most important contributory factors involved in the alteration of microbiome diversity is dietary nutrients (Figure 1). Ingested nutrients are used by the microbes for harvesting energy and basic biological processes. Output of these processes would have significant effects on balancing the bacterial species and host physiology as well [2, 39]. A diet that is high in animal protein affects the diversity of microbiome by enhancing Alistipes spp., Bilophila spp., and Bacteroides spp., whereas it diminishes the beneficial bacteria Roseburia spp., Lactobacillus spp., and E. rectale [40–44]. The results of a study that was conducted on mice showed that high-fat, high-sugar diet caused an increase in Mollicutes and Firmicutes and decrease in Bacteroidetes [40]. In another study, long term high-fat diet led to a considerable decrease in GI barrier-protecting microbiota such as Bifidobacterium spp. which may eventually set out inflammation due to increase in barrier permeability to endotoxins [45]. The diet consisting of high-animal fat increases lipopolysaccharides (LPS) and trimethylamine-N-oxide (TMAO), while it decreases short-chain fatty acid (SCFA) level as an anti-inflammatory factor [42, 44]. Indeed, LPS that is considered as endotoxin through breakdown of the GI barrier may be filtered into lymph, leading to inflammation [40]. Furthermore, LPS through binding to Toll-like receptor 4 (TLR4) which is expressed on many cells and on macrophages as well leads to activation of some pathways that are involved in inflammation induction [46]. In general, a typical Western diet, making up high-sugar, high-fat foods, has been connected to chronic metabolic conditions such as low-grade inflammation, obesity, and metabolic syndrome [44]. On the other hand, the ketogenic diet might have opposite effect on microbiome and could suppress inflammation. This type of diet unlike the Western diet is able to increase Bacteroidetes and decrease Firmicutes [47, 48]. In one study that was conducted on mice, Mediterranean diet, in comparison to Western diet, caused more diversity in microbiome of the study animals. These animals had a higher quantity of Clostridium, Lactobacillus, Oscillospira, and Faecalibacterium and a lower quantity of Coprococcus and Ruminococcus [49]. Similar studies conducted on a human demonstrated result that aligned with animal studies. The subjects who consumed Mediterranean diet for 3 months had significant shift in their microbiome composition and had a higher abundance of Lachnoclostridium, Enterorhabdus, and Parabacteroides with increase in production of SCFAs. In addition, this type of diet led to decrease in the inflammatory cytokines including IL-17, IL-12, CRP, IP-10, MCP-1, and VEGF and increase in levels of IL-10 as an anti-inflammatory cytokine. As a result, Mediterranean diet might decrease the risk of chronic inflammatory diseases [49, 50]. Mice fed with low-fiber diet for a long time showed considerable microbiome alteration and lack of variety as well [51–53]. However, high-fiber, low-fat diets are able to modify the microbial composition, through shifting the microbiome community towards the advantageous bacteria, Bacteroides and Prevotella, while leading to decrease in Firmicutes [54]. Indeed, high-fiber diet compared to low-fiber diet, through increase in microbiota-derived SCFAs such as lactate, succinate, butyrate, acetate, and propionate, has been connected to the inflammation risk reduction [55, 56]. In addition to beneficial aspects on GI barrier and anti-inflammatory effects, SCFAs that are produced mainly by Actinobacteria and Firmicutes through fermentation of dietary fibers and resistant starch participate in gut cellular homeostasis. SCFAs as a fuel for intestine cells have a potential to maintain their typical cell phenotype and homeostasis [57]. This metabolite also exerts its effect by overcoming oxidative stress [58]. SCFAs influence the epigenome of host cells through multiple mechanisms of action and hence influence the growth and functions of the cell as well as modulate the gene expression [59]. In tumor cells, butyrate also brings about activating epigenetically silenced genes like proapoptotic protein belonging to Bcl-2 homologous and cell cycle inhibitors such as P21, both of which are considered as cancer suppressor genes [60]. Transient butyrate treatment enhanced the reprogramming capability of induced pluripotent stem cells derived from a patient with sickle cell disease by diminishing the epigenetic barrier in refractory somatic cells to reprogramming [61]. Sodium butyrate suppresses the growth of endometrial cancer stem cells [62]. Hence, microbes that produce SCFAs through angiogenesis inhibition might be beneficial in the treatment of cancer. In one study, fecal SCFA concentrations of 344 patients with advanced adenomas who have the 5-year history of fiber consumption were compared to the fecal SCFA concentrations of healthy matched individuals as controls [63]. The result showed that fiber intake and levels of fecal SCFAs were significantly lower in patients with colorectal adenoma. Distinct segregation in the microbiota composition of the two groups was also illustrated, and the Streptococcus spp. and Enterococcus were more enriched in the adenoma patients than in normal individuals. On the other hand, the population of high-butyrate-producing bacteria including Roseburia, Clostridium, and Eubacterium spp. was significantly lower in the advanced adenoma group. These results suggested either butyrate deficiency or butyrate-producing bacteria reduction owing to low-fiber intake promotes tumorigenesis [63]. Fecal transplant from mice on high-fat diet reduced expression of tight junction genes in the GI and mammary gland. Moreover, infecting breast cancer cells with microbiome derived from high-fat diet increased proliferation of these cells [64]. The nutrients produced by microbial metabolism of indigestible foods such as SCFAs, vitamins, polyphenols, and polyamines also appear to influence epigenetic mechanisms which in turn serve as a modifier in this regard [4].

## 5. The Effects of Antibiotics on Microbiota

Antibiotics, to which we are more and more exposed, are considered as disturbing factors in the equilibrium of microbiota population, and their long-lasting administration has multiple downsides including rise in antibiotic resistance, increase in susceptibility to infections, the potential to develop allergies, prompting to develop metabolic syndrome, and other chronic disease [65]. Even though some studies have pointed out that antibiotic use only exerts transient effects on the microbiota composition [66], others imply that antibiotics by interfering in microbiome homeostasis permanently disturb this composition and signaling pathways involved in immune system modulation [67, 68]. In other words, uncontrolled usage of antibiotics through microbiome dysbiosis may increase the cancer risk [69–72]. A widespread case control study on patients with different types of cancers established a link between recurrent courses of antibiotics and the risk of cancer development in various organs of human [73]. Prevalence of breast cancer was higher in transgenic mice that were exposed to a combination of ciprofloxacin and metronidazole for a long time [74]. Likewise, in humans, a number of epidemiological studies showed that there are a dose-dependent association between antibiotic exposure and breast cancer incidence [75, 76]. The use of antibiotics not only influences microbiome in health state but also may have an impact on cancer treatment. Result of one study on mice showed that administration of antibiotic could disrupt the equilibrium of the GI microbiota and in turn reduce the antitumor efficacy of 5-FU [77]. These components even impose the function of distant tissues. Indeed, by influencing the level of circulating metabolites, antibiotics harm the human health [78].

## 6. The Crosstalk between Microbiota and Immune System

Since the constitution of the microbial flora occurs in prenatal period along with immune system development and also the gut as the main immunological organ is the primary accommodation of microbiota, it is undeniable that the microbiota could be an important contributing factor in immune response modulation [79]. Studies of GF animals showed that lack of microbiota is linked with strong defect in lymphoid tissue structure of the intestine and its immune functions as well [80]. The microbiota has expansive effects on innate and adaptive immunity at various levels. In the same way, these microbes are able to modulate both local and systemic immune responses of host [81–84]. Pattern recognition receptors (PRRs) as detectors of pathogen components are expressed in the human body by many cells including immune cells. Among PRRs, TLRs are expressed mainly on B cells and macrophages. Microbes mainly cause local immune responses via interactions with this type of receptors [85, 86]. In addition, microbes or their byproducts and metabolites stimulate local dendritic cells (DCs) through interactions with PRRs [82, 87]. These local activated DCs then can travel from their area to mesenteric lymph nodes, triggering the differentiation of naive T cells into T helper 17 (Th17) and regulatory T cells (Tregs) in particular [88]. Again, a subpopulation of these effectors T cells travels back to their original place of residence and regulates local immune responses. Meanwhile, a subset of T cells migrates to the systemic circulation and induces systemic immunity. Th17 cells elicit an extremely inflammatory immune response by secreting cytokines such as IL-17 or by activating neutrophils [89]. Conversely, regulatory T cells as pivotal gatekeepers in immune homeostasis mediate suppression of inflammation to control undesired immune reactions by the engagement of DCs and releasing IL-10 and TGF-*β* [90]. Th17 cells are mostly resident in small intestinal lamina propria, among others. In an interesting study, GF mice without Th17 cells were selected and were colonized by a single subset of bacteria called segmented filamentous (SFB). These commensal bacteria could induce accumulation of Th17 in the intestine. This exciting relationship strongly supports the crucial role of commensal microbes in Th17 cell activation and contributes to protection of human from microbial pathogens [91]. The microbiota community in neonatal intestine modifies the development of Treg population over the first year of life [92]. In GF mice or antibiotic-treated mice, the abundance of Tregs in the lamina propria was significantly reduced, suggesting that the microbiota contributes to Treg differentiation or maintenance [93, 94]. Colonization of GF animals with several strains of Clostridium is adequate to induce Treg in GI [93]. In addition, transfection of Bacteroides fragilis, a human commensal bacterium, led to the induction of Tregs in the mouse [95]. Human microbial composition also impacts on B cells as the main mediator of GI mucosal hemostasis through producing immunoglobulin A (IgA). A number of studies demonstrated that the level of this secretory antibody in GF and newborns animals is significantly low which is reversible upon colonization of microbiota [96].

## 7. The Microbiota and Cancer Development

Mounting evidence emphasized the dual role of the microbiota in conserving health state of host. Commercial microbes are able to protect host's homeostasis via different mechanisms or by producing variable byproducts and metabolites. Conversely, increasing the proportion of some microbiota mainly through producing different toxins contributes toward inflammation, infection, and tumorigenesis.

### 7.1. Microbiome May Reduce Cancer Rate

Host microbiome possesses a variety of functions that can prevent tumor development and progression (Figure 1).

#### 7.1.1. Reinforcement of Mucosal Barrier

The GI epithelium is shielded by protective mucus which is mainly produced by the goblet cells and entraps pathogens and prevents their migration to other tissues. Existence of many immunomodulatory molecules in this layer also illustrates its important role in the immune system [97]. Normal gut bacteria via their byproducts or their effects on immune system are considered as key elements for mucus production, as proved by decrease in number and size of goblet cells and thinner mucosal layer in GF animals in comparison with conventionally raised animals [98, 99]. Commensal microbiomes would derive noteworthy benefit from the power to modify mucus synthesis or secretion from intestinal goblet cells. These modifications contribute to stronger coated layer that prevent enteric pathogens. This defensive strategy may reduce the pathogen-driven cancers [98].

#### 7.1.2. Improvement in the Antitumor Immunity

Through modifying antitumor immunity, microbiota might reduce the power of tumor cells. For instance, beneficial microbiota via regulation of monocytes triggers the NK cell-DC axis in tumor microenvironment [100, 101]. Study on lactic acid bacteria showed that feeding by Lactobacillus rhamnosus, Lactobacillus acidophilus, and Bifidobacterium lactis brought about immune system improvement through increase in the phagocytic activity of peritoneal macrophages and peripheral blood leucocytes in comparison with the control mice. In addition, spleen cells from mice fed with these probiotics unveiled higher NK cell cytotoxic activity compared with untreated cells. Therefore, both natural and acquired immunity responses are modified by lactic acid bacteria [102]. It is also reported that mice colonized by 11 bacterial strains which were isolated from healthy human feces with capability of IFN-*γ* production by CD8+ T cells showed considerable resistance against tumor development. This ability was attributed to human microbiota and their antitumor immunity effects [103]

#### 7.1.3. Reduction in Inflammation

Inflammation has been accepted to play a major role in the pathogenesis of cancer. Some of commensal microbes are capable of modulating tumorigenesis by anti-inflammatory mechanisms. For instance, Escherichia coli KUB-36 which has the potential power to produce seven SCFA elicited anti-inflammatory activity and accordingly inhibited tumor development. Mechanistically, SCFA and other metabolites of Escherichia coli KUB-36 repressed inflammatory cytokines TNF-*α*, IL-6, IL-8, and IL-1*β* as well [104]. Another commensal bacterium which provides anti-inflammatory condition is Faecalibacterium prausnitzii (F. prausnitzii). It is demonstrated that F. prausnitzii exert anti-inflammatory effects through inhibition of NF-*κ*B activation and IL-8 secretion in Caco-2 colorectal cells. Stimulation of peripheral blood mononuclear cells by this microbiome resulted in rising in IL-10/IL-12 ratio [105]. These results are consistent with this fact that living in a GF environment makes individuals more susceptible to pathogens and disease development. When GF mice were transfected by microbial community from wild relative and maintained in laboratory for several generations, they showed a significant reduction in inflammation following influenza virus exposure [106].

#### 7.1.4. Reduction in Systemic Genotoxicity

GF mice transfected by wild microbiota from relative animals provided more resistance against some mutagen factors and survival chance increased among them as well [106]. The beneficial role of particular bacteria against malignancy could be a result of a reduction in systemic genotoxicity as reported in oral inoculation of Lactobacillus johnsonii to B cell lymphoma susceptible mice [107]. Indeed, systemic genotoxicity is mainly created by inflammatory mediators [108] and Lactobacillus johnsonii significantly reduced the level of immune cells such as NK and T cells as well as proinflammatory factors whereas it elevated the anti-inflammatory cytokines. Accordingly, it promoted the clearance of intracellular and systematic genotoxic substances [107].

#### 7.1.5. Activation of Antitumor Signaling Pathways

Emerging evidence has widely reported that the anticancer properties of beneficial microbiota are probably exerted through antitumor signaling activation. For instance, it was demonstrated that P8 as a probiotic-derived protein can be regarded as a novel therapy in colorectal cancer [109]. The involved mechanism in antiproliferative effects of P8 resulted from cell cycle arrest in G2 phase via the p53-p21 pathway. Interestingly, the antiproliferative effect of the endogenous P8 expression was twofold in comparison with exogenous treatment. In mice, oral administration of Lactobacillus acidophilus in colorectal cancer (CRC) could control the growth of the tumor by increasing the apoptosis [110]. In another study, this probiotic inhibited cancer cell proliferation and nudge these cells toward apoptosis via downregulation of NF-kB and MAPK signaling. Lactobacillus reuteri suppressed cell proliferation proteins such as Cox-2 and cyclin D1 and antiapoptotic proteins such as Bcl-2 and Bcl-xL [111]. Secondary bile acids reduced proliferation of breast cancer cells and suppressed aggressiveness of primary tumors by inducing the mesenchymal-to-epithelial transition (MET) [112]. Cell-free Lactobacillus supernatants inhibited the growth of HT-29 colon cancer cells via damage to cell membrane of these cells [113]. Another same study also demonstrated that cell-free supernatant of isolated lactic acid bacteria has anticancer properties on two colorectal cancer cells [114].

### 7.2. Microbiome May Increase Cancer Incidence

While it is well verified that the presence of host microbiota results in mutagen resistance in addition to viral resistance, growing reports imply that certain microbiome is closely associated with the development and progression of various types of malignancy (Figure 1) [115–119]. About 20% of the worldwide cancer burden has been expected to be triggered or modulated by microbes and their byproducts [120]. It is confirmed that Helicobacter pylori is the most important microbe in gastric cancer development [121, 122]. A number of mechanisms have been suggested through which human microbiota contributes to the cancer development such as inflammation generation [31], transfer of tumor-vulnerable phenotype [123], immunosuppression [124], induction of protumorigenic environment [125], and genotoxin accumulation [126].

#### 7.2.1. Inflammation Induction

Most often, it is considered that carcinogenesis functions are secondary to the local long-lasting inflammation, one of the hallmarks of cancer [127]. For instance, carcinogenesis can result from induction of proinflammatory toxins produced by the certain bacterial species such as those produced by Bacteroides fragilis [34, 128, 129]. In dysbiotic states, microbiota affects inflammatory responses through inducing the production of IL-17, IL-1*β*, and IL-23 by *γδ* T cells and myeloid cells [130]. Some species of the Streptococcaceae family in host body such as Streptococcus australis and Streptococcus parasanguinis were correlated with increase in IFN*γ* level, a proinflammatory cytokine [79]. Different inflammatory cytokines provided by special microbiome may damage the DNA in different ways including aberrantly DNA methylation. These DNA damages can trigger tumorigenesis over time [131]. In the presence of commensal microbes, TLR5 induces systematic secretion of IL-6 and trigger inflammation and subsequently accelerates tumor progression [132]. The long-lasting inflammation may also induce dysbiosis and therefore by altering the composition of normal flora and increasing the chance of growth of certain bacteria with genotoxic capabilities provide a suitable environment for tumorigenesis [133, 134]. Nevertheless, certain bacteria, such as H. pylori, also exert direct genotoxic effects on signaling pathways that regulate cell proliferation [121].

#### 7.2.2. EMT

Changes in cell phenotypes through EMT have been revealed to exert an important role in invasion of tumors. As mentioned in the previous section, a main way that dysbiosis triggers cancer is through EMT induction [31]. EMT induction is mainly due to direct pathogen attachment to the mucosal layers and prevention of intercellular adhesions between epithelial cells, high expression of Zeb1 as EMT activators [135], E-cadherin/*β*-catenin signaling activation [136], and binding to E-cadherin as a key protein of the adherent junctions which maintains epithelial phenotype, and its suppression dysregulates cell polarity and downstream signaling pathways [137].

#### 7.2.3. Immunosuppression

Fusobacterium nucleatum (F. nucleatum), a periodontal bacterium enriched in the microenvironment of a number of tumors [138], is able to suppress immune system mainly through NK cell inhibition. In the presence of F. nucleatum, these immune cells were inactivated because of interacting of Fap2 protein of F. nucleatum with an inhibitory NK cell receptor, TIGIT (T cell immunoglobulin and ITIM domain). Since CD4+ memory T cells also express TIGIT, their behavior in the presence of F. nucleatum was tested and the hindering of IFN-*γ* secretion was observed. Therefore, this bacterium exerts immune evasion via binding with immune cell inhibitory receptors [35]. It is shown that patients with pancreatic cancer harbor more microbiome compared with those with normal pancreases. Ablation of this microbiome community from the pancreas decelerated the invasion of tumor because of reprogramming of immune responses. Increase in Th1 differentiation of CD4+ T cells, activation of CD8+ T cell, differentiation of M1 macrophage, and diminution in myeloid-derived suppressor cell infiltration were the results of this depletion [124].

#### 7.2.4. Induction of Protumorigenic Environment and Genotoxin Accumulation

A number of microbial communities living in our body appear to have a role in carcinogenesis and tumor progression by producing detrimental metabolites and factors [117], including production of cytolethal toxin by Campylobacter jejuni [139] and colibactin from E. coli [140]. In colon cancer, results of studies on enterotoxigenic Bacteroides fragilis (ETBF) revealed that this bacterium by producing Bacteroides fragilis toxin (BFT) is able to cleave E-cadherin as a tumor suppressor resulting in the activation of Wnt signaling which in turn increases the expression of MYC as a protooncogene, cell proliferation, and tumorigenesis [141]. Human microbiota may also contribute to tumor angiogenesis, which enables tumor to grow easily. At the tumorigenic site, LPS and unmethylated CpG as two bacterial ligands can activate the host TLRs. Activated TLRs in this condition are able to synthesize, release vascular endothelial growth factor (VEGF), and promote angiogenesis [142]. Human microbiome also promotes angiogenesis and cancer metastasis through their quorum sensing peptides [143, 144]. In fact, metastasis induced by quorum peptides occurs through interaction of these peptides with EGF receptors and in turn stimulates the Ras-STAT signaling pathways. Another possible mechanism by which microbiota contributed to metastasis was attributed to increase in NF-*κ*B that is able to trigger angiogenesis and invasion [144].

### 7.3. The Role of GI Microbiota on Cancer in Distant Organs

Through translocation or altering the metabolism, changes in gut microbiome composition might be associated with tumor development in distant organs such as the breast [145, 146] and liver [147]. A number of studies have established that the GI microbiota of patients with breast cancer changes compared to that of healthy matched women [148]. Several studies showed that the metabolism of estrogen as the most important hormone involved in breast cancer can be affected by environment features of GI [149]. Increase in conjugated estrogens in fecal of individuals who used ampicillin underscores the role of microbiota in metabolism of this hormone [150]. Two types of GI bacteria include Helicobacter specie [151] and Salmonella typhi [152] known as oncogenic microbial involved in hepatocellular carcinoma and gallbladder cancer, respectively. Results of a study showed that treatment of gastric adenocarcinoma patients with specific antibiotics that are known as a standard in H. pylori elimination also resulted in the remission of mucosa-associated lymphoid tissue (MALT) lymphoma in low grade. Identification of H. pylori as a class I carcinogen also illustrates this claim [121, 122].

## 8. Microbiome Can Influence Anticancer Therapies

Chemotherapy and immunotherapy fail to show efficacy in pathogen-free animals [153, 154]. This opinion has been confirmed in mice with the absence of specific bacterial strains involving in expansion of immune system [155, 156]. That is to say, commensal microbiota and their diversity are required for a functional host immune system in response to anticancer compounds [153, 154]. Intact microbiota is able to regulate the tumor microenvironment via modulating the tumor-infiltrating myeloid cells. Hence, when used along with anticancer component, intact microbiota may improve the response of chemotherapy or immunotherapy. It is considered that microbiota may modify response of patients to treatment by modulating the microenvironment of tumor [154]. Cyclophosphamide (CTX) is known as one of the immunomodulatory compounds. When two gut commensal bacteriomes, Barnesiella intestinihominis and Enterococcus hirae, were used in the context of CTX, patients showed more promising response in comparison with patients who received only CTX. Mechanistically, these two bacteria modified the immune system via increasing the ratio of cytotoxic T cells/T regulatory cells in tumor environment [157]. Results of one study showed that CTX may exert its toxic effect on tumors through altering microbiota composition in the intestine. Indeed, this component triggers the translocation of distinct gram-positive bacteria (primarily Lactobacillus johnsonii and Enterococcus hirae) into secondary lymphoid tissues which in turn stimulates the TH17 and TH1 generation and tumor suppression, whereas lack of these specific bacteria caused resistance to CTX [155]. Certain GI bacterial species including Bacteroidales, Burkholderiales, and Bifidobacteriales orders by affecting tumor microenvironment modified the effectiveness of anti-CTLA4 or anti-PDL-1, respectively [156, 158]. Platinum chemotherapy and CpG-oligonucleotide immunotherapy via ROS production can cause DNA damage in cancerous cells which resulting in prolonged survival in patients. Prescription of these two types of drugs in germ-free mice with subcutaneous tumors revealed a significant decrease in tumor degeneration and animal survival in comparison with intact commensal microbiota condition [154]. The cytotoxic effects of camptothecin, podophyllotoxins, alkylating agents, and anthracyclines on cancerous cells through ROS production accentuate the undeniable role of the host microbiota in the optimal response to treatment [159–162].

## 9. Fecal Microbiota Transplantation: An Innovative Treatment for Cancer

The microbiota via a range of proposed mechanisms is able to influence response of cancer to conventional treatments including chemotherapy and immune checkpoint inhibitors (CPIs). Based on this evidence, fecal microbiota transplantation (FMT) has been proposed as an excellent method of cancer management. For the first time, an ancient Chinese physician of the fourth century used the fecal microbiota of healthy individuals as a remedy for patients with severe diarrhea and achieved beneficial results [163]. In 1958, in patients with Clostridium difficile infection who were resistant to antibiotics, FMT was administrated [164]. Owing to promising response, FMT has been evolved as a new treatment option for this infection since 2013. Patients who received FMT as treatment showed a higher response rate (81%) in comparison with those who used antibiotics (31%) [165]. The beneficial role of FTM in metabolic syndrome and colitis was also demonstrated [166, 167]. Since then, utilizing FMT through different routes such as oral capsules, nasogastric tube, enema, and colonoscopy in different clinical settings has been growing. There is a controversy about response rates of variable route administration. For instance, administration via colonoscopy or enema showed higher response compared to other paths [168]. However, in another clinical study, there was no longer difference between two routes of colonoscopy and encapsulated components [169]. Cancer immunotherapy is considered as a major breakthrough in the fight against cancer. CPIs have been placed at the first line of cancer immunotherapy, mostly due to its widespread bioactivity among different cancer types and effectiveness against metastatic tumors [170]. Programmed cell death 1 (PD-1), programmed cell death ligand 1 (PD-L1), and cytotoxic T lymphocyte-associated protein 4 (CTLA-4) are the most well-known immune checkpoints, which suppress T cell activation and, therefore, diminish immune responses against cancer. These unfavorable effects go into reverse by using CPIs, but unfortunately, a minority of patients benefit from anticancer effects of CPIs [171]. In addition, over time, cytotoxic drugs including CPIs can cause unpredictable side effects. That is why immunotherapy has become a major issue of concern. Since it is considered that the intestinal flora is a strong modulator of immune responses, this hypothesis that alteration of components of intestinal bacteria may influence the responses to immunotherapy evolved [172]. Initial reports in this regard showed that animals that had received certain bacteria via oral gavage or FM exhibited greater sensitivity to CPIs [156]. Subsequent investigations pronounced a relation between the human microbiota profile and CPI responses [153, 173]. Recently, two groups of researchers showed that manipulating the gut microbiota may enable patients to triumph over CPI resistance. Baruch et al. selected 10 melanoma patients who failed to respond to CPIs. These patients were subjected to FMT from donors who had showed a complete response to CPIs. In order to deplete the gut microbiota, eligible recipients were treated with antibiotics before FMT administration. After that, these patients were administrated FMI every 14 days via colonoscopy along with CPI treatment (nivolumab) in standard dose. Among these 10 recipients, the size of tumors shrank in 3 patients. In addition, treatment with FMT leads to an encouraging change in infiltration of immune cells and expression of favorable immune-related genes in both tumor environments and gastrointestinal tract [174]. Recipient patients who were selected by Davar's group had progressive melanoma and showed complete resistance to CPI therapy prior to FMT administration. Stool samples were selected from seven patients who responded to CPIs completely or partially. The recipients were treated with single FMT in combination with pembrolizumab as a CPI every 3 weeks. The results of their study showed that 6 out of 15 patients provided clinical benefits. Indeed, FMT administered via colonoscopy reprogrammed the microenvironment of tumor by changing the gut microbiota composition in patients with PD-1 refractory melanoma that consequently improved the anti-PD-1 responses [175]. Promising results of Baruch et al. and Davar et al. not only suggest the effectiveness of FMT in PD-1 refractory patients but also incentivize researchers to conduct more studies to address numerous scientific questions in this respect.

## 10. The Role of Microbiome Signature in Cancer Diagnosis

The microbiota signature is emerging as a novel diagnostic and prognostic clinical method for the management of cancer. Mounting evidence underscores that dysbiosis in microbiota could be a noninvasive tool to the detection of a range of GI malignancies in early stage. For instance, the toxin-producing bacteria ETBF is considered to induce colorectal carcinogenesis by altering the mucosal immune response and inducing epithelial cell alterations. Results of one study demonstrated that in addition to a significant level of concordance in terms of ETBF between different colonic locations (86%), there might be strong associations between ETBF positivity and the occurrence of tubular adenomas, low-grade dysplasia, and serrated polyps. Hence, it was proposed that fecal recognition of ETBF may be a diagnostic marker of colorectal cancer in early stage [34]. Two clinical trials published recently looked into the possibility of relationship between fecal bacteria markers and gastric cancer (GC) risk in healthy people. Liu's group suggested that detection of Desulfovibrio, Escherichia, Faecalibacterium, and Oscillospira as fecal biomarkers could precisely predict GC with an accuracy of over 90% [176]. Evaluation of sequencing data regarding GI microbiota belonging to patients with esophageal cancer (EC) showed that the intestinal microbiota in patients with this type of cancer was significantly higher than normal persons. Furthermore, aside from discrimination that was recognized in gut microbiota profile between EC and normal persons, Lachnospira seems to be accurate potential biomarker in EC diagnosis [177]. Interestingly, even though 70% of GC is attributed to Helicobacter pylori infection, this bacterium is not a useful screening sign. It is because only 1–4% of the population who harbor this bacterium will develop GC [176, 178]. However, the gut Helicobacter pylori has been considered as a carcinogenesis factor in hepatocellular carcinoma (HCC) due to the gut–liver axis [179, 180]. A higher proportion of fecal E. coli in patients with hepatic cancer than the healthy individual contributes to the early diagnosis of disease and provides opportunities for the development of an effective preventive measure [181]. Investigation into fecal samples of a large number of patients showed that diversity of microbial composition was higher in early HCC compared to cirrhosis [182]. Several clinical studies conducted to explore the role of microbiota in pancreatic adenoma or pancreatic cancer incidence revealed that oral, fecal, and pancreatic microbiota communities in these patients are different from those in healthy individuals [183–185]. The comparison between the fecal microbiota of patients with pancreatic ductal adenocarcinoma (PDAC) and normal individuals represented a sharp rise in Bacteroidetes abundance and a significant decrease in Firmicutes and Proteobacteria abundance [186]. In addition to GI cancers, dysbiosis of intestinal microbiota has been reported in patients who suffer from a non-GI cancer. This imbalance was verified in breast cancer with an increase in abundance of Faecalibacterium, Ruminococcaceae, and Clostridiaceae along with a decline in the abundance of Lachnospiraceae and Dorea compared to normal persons, hence suggesting that the GI microbiome could be viewed as a diagnostic biomarker in breast cancer [148]. The results of one study where the GI microbiota of 30 patients with lung cancer was compared to 30 healthy persons showed a significant difference between their gut microbiome. It means that while the levels of Enterococcus were elevated in these patients, the richness of phylum Actinobacteria and genus Bifidobacterium was higher in the control group [187]. Circulating metabolites of microbiota could also be used to diagnose cancer. Using 16S rDNA gene sequencing, the difference between the GI microbiome composition of children and teenagers with acute lymphoblastic leukemia and their healthy siblings was recognized either before or during chemotherapy [188]. Accordingly, microbiome signature can be used as a predictor of infection risk in patients with ALL diagnosis. Evaluation of the gut microbiome before chemotherapy may help design treatment regimens to modify gut dysbiosis and alleviate infection risk during chemotherapy [189]. Nonetheless, practical microbiome signature at the metabolomics or metagenomic level fails to be used in clinic as a cancer screening method and more forthcoming studies are required to prove whether these biomarkers can accurately recognize patients at risk for malignancy to improve disease diagnosis and management.

## 11. The Effect of COVID-19 on Microbiota

Among different kinds of pathogenic infections, those caused by viruses are the most serious challenge that the public health system is facing. Apart from detrimental effects on the host body, viruses may have significant effects on the commensal microbiota. Mounting evidence emphasizes this proposal and suggested that commensal microbiota may have stimulatory or suppressive roles in viral infections through diverse mechanisms. Microbiota homeostasis plays an imperative role in protecting the host against various viruses [190]. However, a great deal of evidence demonstrated that viral infections can disturb homeostasis in microbiota which in turn develops pathogenic condition. Increase in Enterobacteriaceae in the lungs and drop of Lactococci and Lactobacilli in the intestine as a result of exposure to influenza virus are a typical example to illustrate the point. The world has been facing a global pandemic owing to the outbreak of a new coronavirus called coronavirus disease 2019 (COVID-19), a severe contagious disease caused by severe acute respiratory syndrome coronavirus 2 (SARS-CoV-2). Even though our knowledge about the relationship between the microbiota composition and cancer in the presence of SARS-COVID-19 is not complete, growing evidence from different studies in this respect will pave the way to find a feasibly new strategy for controlling this infection and its side effects in the future. A meta-analysis study has showed that over 20% of patients with COVID-19 suffer from GI symptoms [191]. Other studies reported that SARS-CoV-2 was detected in stool samples and anal swabs of 50% of these patients [192, 193]. As GI is the most important organ in which microbiota is settled, these findings may suggest that there is a strong interaction between microbiota and SARS-CoV-2. Furthermore, as the special receptor for COVID-19, the angiotensin-converting enzyme 2 (ACE2) receptor not only is expressed highly in GI but also contributes to controlling inflammation of this organ [194–196]. Further results showed that this virus is able to disturb the homeostasis of microbiota, which is characterized by augmentation of opportunistic commensal microbes and depletion of beneficial microbes. This dysbiosis even remained after clearance of virus suggesting that COVID-19 infection may result in long-lasting damaging effect on the body such as cancer development [191]. Regarding immune system regulation, some gut bacteria that are known as immunomodulatory potential bacteria such as Eubacterium rectale and Faecalibacterium prausnitzii and several bifidobacterial species were depleted in patients with COVID-19 [197]. Results of a cohort study showed that diminution of some bacterial species in patients with COVID-19 was connected to increase in the level of IL-10, TNF-*α*, CXCL10, and CCL2 and immunological responses [198]. The association between the gut microbiome composition and severity of disease and level of inflammatory cytokines, chemokines, and markers of tissue damage in patients with COVID-19 is also illustrated. For instance, patients with COVID-19 who were admitted in the intensive care unit (ICU) showed a higher abundance of main proinflammatory cytokines, such as TNF*α*, IL-2, IL-7, IL-10, MCP1, MIP1A, G-CSF, and IP10 in comparison with non-ICU patients [199]. These cytokines were apparently associated with a specific pattern of the GI microbiome [79]. Future work in this respect will help to develop new strategy in managing and controlling viral infections within the complicated environments of the human body. Apart from the direct interaction between the virus and the host microbiota composition, the lifestyle that people have adopted during this pandemic period, including wearing masks, washing hands and materials repetitively, disinfecting surfaces, social distancing and being away from the population, and lack of interaction and communication with others, should be taken into consideration as a threat to microbiota homeostasis. This excessive hygiene is capable of altering the microbiota composition and in such way impacting the immune system and disturbing our potential to fight against pathogens, all of which are considered as risk factors for cancer development.

## 12. Conclusion

As human microbiome composition and their metabolites influence many features of human physiology, it appears logical, then, that they may impact cancer prevention, treatment, and management. This possibility is underscored by promising results that are achieved regarding the efficacy of microbiome-based therapy. The balance of microbiota community is influenced by a variety of factors including genetic and environmental variations. Therefore, it is important to consider that adopting a healthy lifestyle helps people to maintain this microbiota equilibrium and accordingly decreases the rate of pathologic condition rate. Even though the association between the microbiota and various cancers has been verified, far more research should be carried out to fully untangle this complex network. For example, it is crucial to know what and how various factors and pathogens influence microbiota community, the accurate molecular pathways through which the microbiota impacts oncogenes and antioncogenes. The potential ability of microbiota to either modify or interfere with the cancer therapy might be another area of research.
